# Evaluating the impact of screening plus eave tubes on malaria transmission compared to current best practice in central Côte d’Ivoire: a two armed cluster randomized controlled trial

**DOI:** 10.1186/s12889-018-5746-5

**Published:** 2018-07-18

**Authors:** Eleanore D. Sternberg, Jackie Cook, Ludovic P. Ahoua Alou, Carine J. Aoura, Serge Brice Assi, Dimi Théodore Doudou, A. Alphonsine Koffi, Raphael N’Guessan, Welbeck A. Oumbouke, Rachel A. Smith, Eve Worrall, Immo Kleinschmidt, Matthew B. Thomas

**Affiliations:** 1 Department of Entomology and Center for Infectious Disease Dynamics, The Pennsylvania State University, University Park, 16802 PA USA; 2 MRC Tropical Epidemiology Group, Department of Infectious Disease Epidemiology, London School of Hygiene and Tropical Medicine, London, UK; 3 Institut Pierre Richet (IPR) / Institut National de Santé Publique (INSP), Bouaké, Côte d’Ivoire; 4 Université Alassane Ouattara, Bouaké, Côte d’Ivoire; 5 Department of Disease Control, London School of Hygiene and Tropical Medicine, London, UK; 6 Department of Communication Arts and Sciences and Center for Infectious Disease Dynamics, The Pennsylvania State University, University Park, 16802 PA USA; 7 Department of Vector Biology, Liverpool School of Tropical Medicine, Liverpool, UK; 8 School of Pathology, Faculty of Health Sciences, University of the Witwatersrand, Johannesburg, South Africa

**Keywords:** Eave tubes, Screening plus eave tubes, SET, Housing, House screening, Malaria control, Clinical malaria, LLINs, Cost-effectiveness, Economic evaluation

## Abstract

**Background:**

Access to long-lasting insecticidal nets (LLINs) has increased and malaria has decreased globally, but malaria transmission remains high in parts of sub-Saharan Africa and insecticide resistance threatens current progress. Eave tubes are a new tool for the targeted delivery of insecticides against mosquitoes attempting to enter houses. The primary objective of this trial is to test whether screening plus eave tubes (SET) provides protection against malaria, on top of universal coverage with LLINs in an area of intense pyrethroid resistance. The trial will also assess acceptability and cost-effectiveness of the intervention.

**Methods/design:**

A two-armed, cluster randomized controlled trial will be conducted to evaluate the effect of SET on clinical malaria incidence in children living in central Côte d’Ivoire. Forty villages will be selected based on population size and the proportion of houses suitable for modification with SET. Using restricted randomization, half the villages will be assigned to the treatment arm (SET + LLINs) and the remainder will be assigned to the control arm (LLINs only). In both arms, LLINs will be distributed and in the treatment arm, householders will be offered SET.

Fifty children aged six months to eight years old will be enrolled from randomly selected households in each of the 40 villages. Cohorts will be cleared of malaria parasites at the start of the study and one year after recruitment, and will be monitored for clinical malaria case incidence by active case detection over two years. Mosquito densities will be assessed using CDC light traps and human landing catches and a subset of *Anopheles* mosquitoes will be examined for parity status and tested for sporozoite infection.

Acceptability of SET will be monitored using surveys and focus groups. Cost-effectiveness analysis will measure the incremental cost per case averted and per disability-adjusted life year (DALY) averted of adding SET to LLINs. Economic and financial costs will be estimated from societal and provider perspective using standard economic evaluation methods.

**Discussion:**

This study will be the first evaluation of the epidemiological impact of SET. Trial findings will show whether SET is a viable, cost-effective technology for malaria control in Côte d’Ivoire and possibly elsewhere.

**Trial registration:**

ISRCTN18145556, registered on 01 February 2017 – retrospectively registered.

## Background

The incidence of malaria worldwide declined by 41% between 2000 and 2015 [1, 2]. Malaria vector control tools, primarily long-lasting insecticidal nets (LLINs) and indoor residual spraying (IRS), have been instrumental in reducing the global disease burden [2]. Maintaining and extending this progress requires innovative, cost-effective vector control methods to overcome challenges including remaining areas of high transmission, increasing levels of insecticide resistance in vector populations, and operational and financing constraints on LLIN and IRS [3–8].

Although house modifications to prevent mosquito entry are not a new malaria control tool [9, 10], there is recent renewed interest in improved housing - such as replacing thatch roofs with metal, screening windows, and closing eaves (i.e., the gap between the top of the wall and the roof) - for the purpose of malaria control [11]. An ongoing randomized controlled trial (RCT) in the Gambia is testing the epidemiological and entomological impact of replacing thatch roofs with metal, and screening open eaves, windows and doors, in addition to the current best practice of LLINs [12]. Another RCT carried out in the same region found a 22% reduction in the number of malaria vectors (*Anopheles gambiae* mosquitoes) captured indoors, and a 7% reduction in anemia in children living in houses with screening and closed eaves, compared to unmodified houses [13]. A recent detailed analysis of survey data from 21 countries throughout sub-Saharan Africa found a significant inverse association between “modern” house traits and reduced malaria prevalence in children, on par with the protection provided by insecticide treated bednets [14].

Open eaves, which are found in many traditional African houses, are a particularly important source of attractive cues for malaria mosquitoes, and represent the key entry point for *An. gambiae* in sub-Saharan Africa [15–18]. It was this aspect of mosquito behavior that motivated the initial development of eave tubes [19–22]. Eave tubes provide targeted insecticide treatment and, in combination with “mosquito proofing” of the house (screening windows, closing eaves and sealing any other cracks or openings), reduce mosquito entry into the house while still maintaining airflow through the eaves. Relying on cues that typically draw malaria mosquitoes into houses, eave tubes turn the house into what is effectively a “lure and kill” device.

The eave tube consists of a section of pipe fitted into a closed eave, with a screened insert that is placed inside the tube. The insert is treated with an electrostatic coating, which holds powder formulations of insecticides. This insecticide delivery method can be highly effective, even against resistant mosquitoes [23]. Killing mosquitoes as they attempt to enter houses via the eave tubes offers potential for community level impact, similar to that observed with insecticide treated bednets, if the coverage of the technology is high [19]. The screening component, which physically reduces mosquito entry into the house, offers passive protection for every person sleeping inside the house, regardless of whether they are under a bednet.

The aim of the cluster randomized controlled trial (CRT) presented here is to determine whether screening plus eave tubes, the SET intervention, provides protection against malaria, on top of the current best practice of universal coverage of LLINs (one bednet per two people). LLINs are the primary tool used for malaria vector control globally [1] and therefore the trial is designed to reveal any benefits from SET on top of standard control measures. The trial will be based in central Côte d’Ivoire, where there is intense insecticide resistance [24], to evaluate the efficacy, acceptability, and cost-effectiveness of the intervention in an area where existing vector control tools may be compromised by resistance.

## Study objectives

### Epidemiology

#### Primary objective

To assess whether SET (screening plus eave tubes) and LLINs (at universal coverage) reduces the incidence of clinical malaria in children between the ages of six months and ten years, compared to universal coverage of LLINs alone.

#### Secondary objectives

- Assess whether SET + LLINs reduces the incidence of malaria infection in children between the ages of six months and ten years, compared to LLINs alone.
- Assess whether SET + LLINs reduces the prevalence of anemia in children between the ages of six months and five years, compared to LLINs alone.
- Assess whether SET + LLINs is associated with a rise in symptoms of respiratory infections in children between the ages of six months and ten years, compared to LLINs alone.

### Entomology

#### Primary objective

Assess the impact of SET + LLINs on indoor and outdoor densities of malaria vectors, compared to LLINs alone.

#### Secondary objectives

- Assess the impact of SET + LLINs on entomological inoculation rate (EIR; mean number of sporozoite infective bites/person/year), compared to LLINs alone.
- Assess the impact of SET + LLINs on parity in the malaria vector population, compared to LLINs alone.
- Monitor levels of insecticide resistance in villages that receive SET + LLINs, compared to villages that receive LLINs alone.

### Social science

#### Primary objective

Assess end users’ perceptions of and willingness to adopt SET in the treatment and control arms, and predictors of those perceptions and actions.

#### Secondary objectives

- Assess malaria prevention behavior in households with SET versus LLINs alone, as well as within the treatment and control arms.
- Assess willingness to maintain SET and identified key factors that influence willingness to maintain.
- Measure attitudes, emotions, knowledge, and beliefs relating to malaria and malaria control interventions, including SET and LLINs.

### Economics

#### Primary objective

Measure the incremental cost-effectiveness of adding SET to LLINs from the societal and provider perspective under trial conditions.

#### Secondary objectives

- Establish the relative contribution to costs of the distinct programmatic elements (house modifications, installation of the eave tubes, treatment and retreatment of the eave tube inserts with insecticides, and maintenance of the housing repairs and eave tubes), and identify the inputs that contribute the most to overall costs.
- Estimate the potential cost of providing SET at larger scale over an extended period of 3 and 5 years under operational scenarios.

## Methods/design

### Study area

**Fig. 1:**
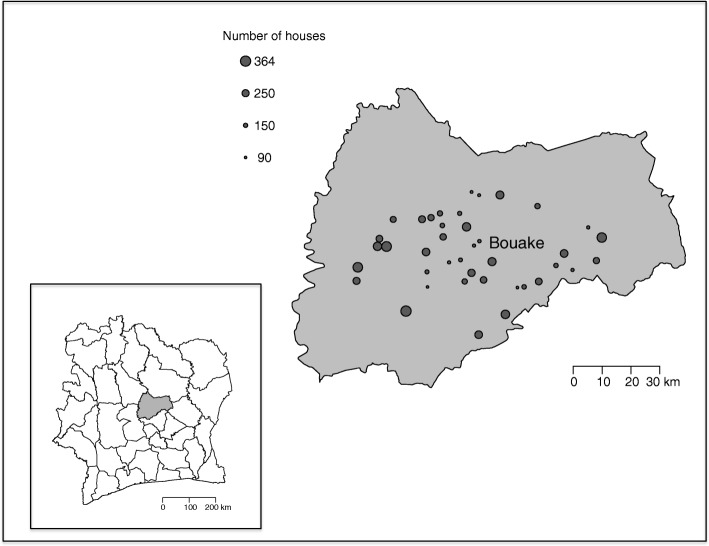
Map of study area and 40 candidate study villages

The study site is situated in the Gbêkê region in central Côte d’Ivoire. It is highly malaria endemic with year-round transmission, peaking during the rainy season (May through October) [25–27]. Forty candidate villages have been identified within a 60 km radius around the town of Bouaké (Fig. 1). Selection criteria for these candidate villages were: between 100 to 600 houses, at least 80% of houses suitable for installation of the SET intervention (i.e. roofs made out of metal sheeting and walls made out of concrete or brick), and villages at least 2 km from any other candidate study village.

### Participant recruitment

Community consent to participate in the study will be obtained through meetings with village leaders and inhabitants. A census will be carried out prior to the start of the study. LLIN distribution numbers will be calculated based on data from the census. Consent for the house modifications will be obtained from individual homeowners during door-to-door visits, following the randomization of villages to trial arms.

Each candidate village is associated with a health center that is responsible for providing medical care to a group of villages. In cases where the health center is not situated in the village, a Community Health Worker (CHW) is charged with providing routine care for uncomplicated malaria, diarrhea, and mild respiratory infections in children. Study team members will visit each of the health centers associated with a trial village, to inform the health center officials of the trial activities. CHWs in the trial villages will be trained in the trial procedures.

Eligible children will be randomly selected for recruitment into the active case detection (ACD) cohort in each village. Recruitment will be limited to children aged eight or younger, to avoid children aging out during the two year monitoring period. Study team members will seek informed consent from the parents or caregivers of the children before enrolling the child in the cohort. Children aged eight will be asked to assent, in addition to parental consent. Children in the treatment arm will be recruited for the cohort irrespective of whether they live in a house that has been modified with SET to avoid bias related to house structure.

### Study design

**Fig. 2:**
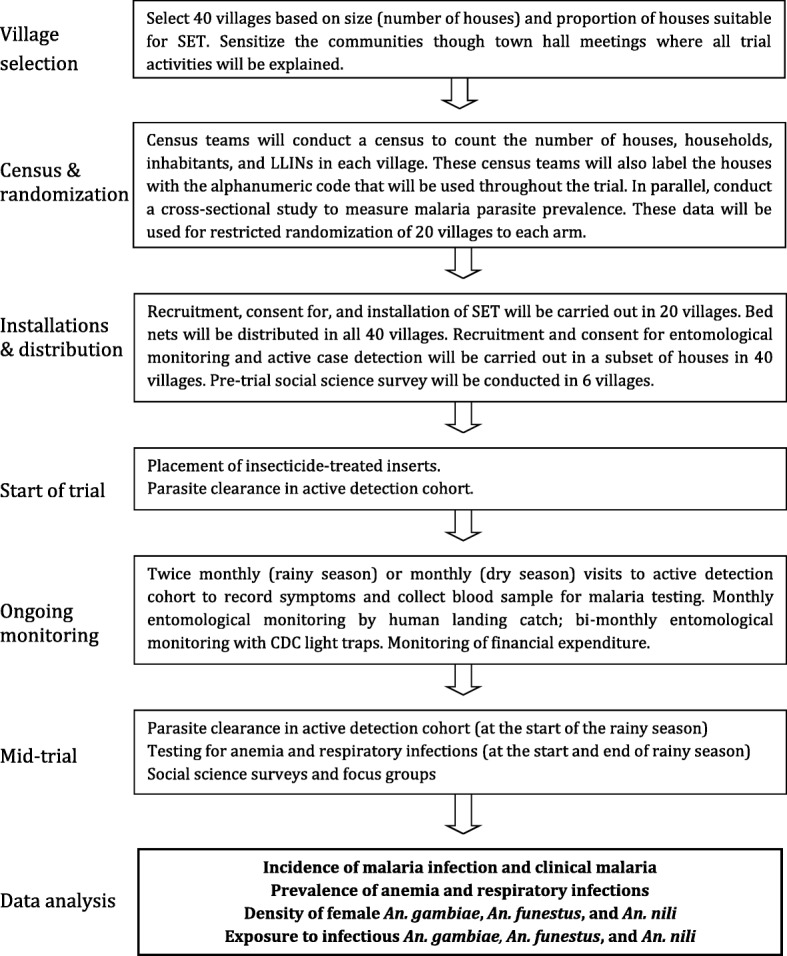
Summary of study design

The study design is summarized in Fig. 2 and a schedule of trial activities is presented in Table 1. The design is a two-armed CRT with 20 villages (clusters) per arm. Villages will be allocated to an arm through restricted randomization. Villages in the control arm will receive universal coverage of LLINs, while the villages in the treatment arm will receive universal coverage of LLINs and heads of household living in suitable houses will also be offered the SET intervention free of charge. The target coverage for SET in the treatment arm is ≥65% of all houses in the village. After the installation of SET (in the treatment arm) and the distribution of LLINs (in both arms), the epidemiological and entomological monitoring will begin.

**Table 1:**
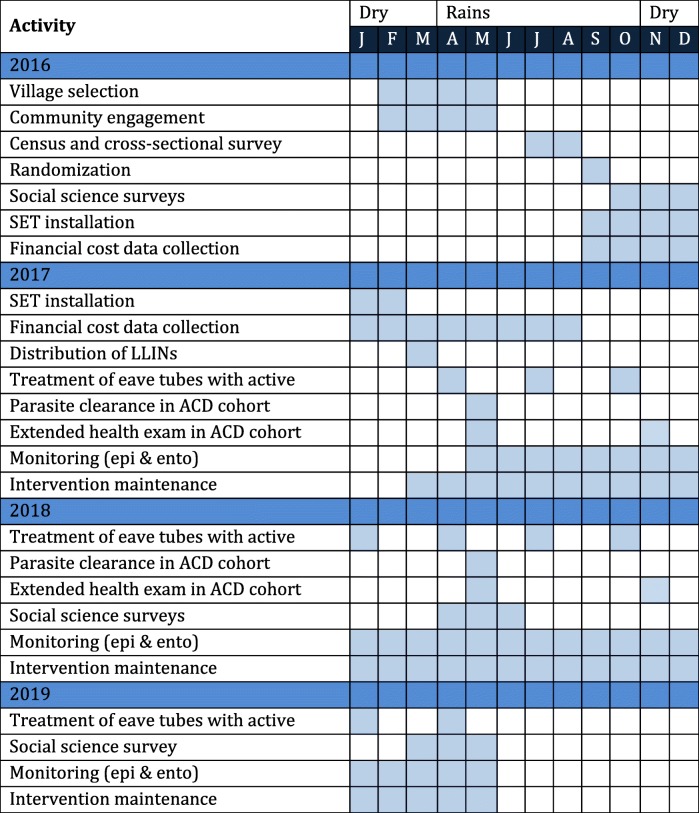
Timetable of trial activities

During the first visit and one year after recruitment, all children in the ACD cohort will be cleared of malarial parasites. Children in the cohort will be monitored for two years to determine clinical malaria incidence, malaria infection incidence, and prevalence of acute respiratory infections. Twice a year, at the start and end of the rainy season, all children in the cohort aged five years or younger will be tested for anemia. If the child leaves the village permanently or the parents withdraw their consent, another child will be recruited into the cohort, preferably of similar age and in the same household.

Indoor mosquito density will be monitored every other month in 12 randomly selected houses in each village using CDC light traps. Indoor and outdoor mosquito density and exposure to infectious bites will be assessed using human landing catches (HLC) every month in four randomly selected houses per village. Four villages in each study arm will be selected for insecticide resistance monitoring. The villages will be selected based on the presence of permanent breeding sites. Mosquitoes from these eight villages will be sampled once a year over four years (once before the start of the study, once each year of the study, and once after the end of the study).

Questionnaires will be administered to consenting participants in six study villages (three in each trial arm) at the start and end of the monitoring period, and in two study villages (one in each trial arm) at the midpoint of the monitoring period. Ethnographic studies will take place in three of the villages in the trial arm during the installation of the SET intervention. Focus groups also will be conducted midway through the trial in two villages from the treatment arm and one village from the control arm. Data from the social science activities will inform the economic evaluation, but no primary cost data will be collected from households.

### Randomization and blinding

Allocation of villages to study arms will be restricted to randomizations that limit the difference between the mean values in each study arm of the following: malaria prevalence; socioeconomic status; village size, and proportion of houses suitable for SET. Data on these variables by cluster will be obtained through a pre-trial cross-sectional survey. Data on any additional potentially confounding ecological factors not included in the randomization will be collected and corrected for in the analysis.

Given the nature of the intervention, it is impossible to conduct this study in a fully blinded manner but those parts of the data collection that can be blinded will be. Observer bias will be reduced where feasible. All laboratory work with samples will be blinded where possible. Standard light traps that do not rely on the ability of the fieldworker to collect specimens will be used to reduce mosquito collector bias, in addition to HLCs. All analyses will be conducted on blinded data.

### Interventions

The SET intervention consists of four elements:

1. House modification. Any open eaves or gaps in the walls will be sealed with concrete. Windows will be screened and damaged doors will be repaired with wood.
2. Eaves tube installation. A 20 cm long piece of PVC pipe with a diameter of 15 cm (the eave tube) will be installed at 1.5 - 2 m intervals into the outer walls of occupied rooms (e.g. bedrooms and living rooms but not storage rooms). The target location will be approximately 20 cm below the roof, but this may need to be adapted depending on different house designs.
3. Maintenance. The condition of the SET intervention will be monitored through village ‘walk-throughs’ every two months, when any damage to window screening, eave tubes, doors, or walls will be recorded and repaired.
4. Insecticide treatment and retreatment. The eave tube inserts will be treated using a commercially available, 10% wettable powder formulation of the pyrethroid insecticide beta-cyfluthrin (Tempo Ultra WP, Bayer). Inserts in the study will be replaced with freshly treated inserts if bioassay mortality falls below 70%.

Persistence of the insecticide will be monitored by monthly bioassays with local, field-collected mosquitoes. These bioassays will consist of an eave tube with the insert placed at the end of the tube. A bottle filled with hot water and covered with a worn sock will be placed behind the insert to act as a host cue. Local, field-collected 2–5 day old female *An. gambiae s.l.* mosquitoes will be allowed to recruit freely to the insert for one hour and knock down and mortality will be assessed 24 h post exposure.

LLINs (Permanet 2.0; Vestergaard Frandsen, Switzerland) will be distributed at universal coverage to each household in all of the study villages. Côte d’Ivoire’s national malaria control program (NMCP) guidelines will be followed to encourage consistent and correct use of the LLINs. During the routine visits to villages for epidemiological and entomological monitoring, trial staff will administer standardized questionnaires, including questions on the presence of LLINs in the sleeping areas and their use by the household.

### Epidemiological evaluations

Children enrolled in the ACD cohort will receive routine visits from the study team (a trained nurse and the CHW) every two weeks (rainy season) or every month (dry season).

At the first visit, additional data will be collected on household level socioeconomic indicators, methods of vector control used in the household, and sleeping habits. Children five years of age or younger will have a blood sample taken for immediate measurement of hemoglobin levels using a spectrophotometer (HemoCue Hb 201+, Radiometer Medical Aps, Ängelholm, Sweden). All children will receive a three-day course of a first-line antimalarial recommended by the NMCP in Côte d’Ivoire (artesunate-amodiaquine or artemether-lumefantrine), to clear any existing malaria parasite infection. The study team will observe adherence for the first dose and parents or guardians will be instructed to complete the treatment at home. The CHW will be instructed to check compliance and the parents or guardians will be asked to bring the empty drug packet to the following visit. A thick blood smear will be taken on the next visit (two weeks later) to confirm parasite clearance.

At all visits, the child’s axillary temperature will be recorded. If the child is febrile (axillary temperature ≥ 37.5 °C), or has a history of fever in the past 48 h, or if the parents report that the child is sick, a health exam will be carried out and a record will be made of the child’s symptoms, pulse, and respiratory rate. A blood sample will be taken from febrile children by finger prick for a malaria rapid diagnostic test (mRDT; SD Bioline Malaria Ag P.f./Pan, Standard Diagnostics, Inc., Gyeonggi, Republic of Korea). A thick blood smear and a blood spot on filter paper will also be taken for subsequent confirmation of the infection. If the mRDT is positive and the study nurse diagnoses uncomplicated malaria, the child will be treated with a first-line antimalarial (artesunate-amodiaquine) for three days, according to the guidelines of the NMCP. Treatment for malaria will be provided free of charge through this system. If the child exhibits any symptoms of severe illness, he or she will be sent immediately to the closest health clinic for treatment. Once a month, a thick blood smear and blood spot will be taken from all children in the cohort to monitor for asymptomatic parasite infections. During these visits, a record will be made of recent travel, and whether the child slept under an LLIN the preceding night.

Four times during the course of the monitoring period, at the start and end of the rainy season, all children in the cohort will be checked for respiratory symptoms (cough, nasal discharge, rales, elevated age specific respiratory rate, and chest indrawing) and children five years of age or younger will have a blood sample taken for immediate measurement of hemoglobin levels using a spectrophotometer. One year after the initial parasite clearance, at the start of the next rainy season, all of the cohort children will again be cleared of malaria parasites with a three-day course of front-line antimalarials (artesunate-amodiaquine), confirmed by thick blood smear on the following visit.

To capture any malaria cases that occur between cohort visits, the parents or guardians will be given a card at the initial visit, identifying the child as a participant in the study, with instructions to present this card whenever the child receives medical treatment between visits. The back of the trial card will provide space to record the date of visit and the diagnosis.

### Entomological evaluations

Each month, mosquitoes will be sampled using human landing catches (HLC) both indoors and outdoors for one night at four randomly selected houses in each of the 40 study villages. Starting at 18:00, one capturer will sit inside of the house in the living room area and one will sit outside of the house. These capturers will collect mosquitoes on their bare feet and legs using small glass tubes, which will then be plugged with cotton and brought back to the laboratory. A supervisor will pass by every hour to ensure that the capturer is awake and following the correct capture protocol. At 01:00, a second team will take over and continue the captures until 08:00. When the HLC samples are brought back to the lab, the mosquitoes will be identified using a species key based on morphological traits [28]. A subset of the captured *An. gambiae s.l.* and all *An. funestus* and *An. nili* females will be dissected to determine parity. Both species are known to be vectors of malaria in Côte d’Ivoire [29–31], however *An. gambiae s.l.* is the numerically dominant vector in the trial area [32]. After dissection, mosquitoes will be preserved for later PCR analysis to determine sporozoite prevalence. PCR will also be used to type *An. gambiae s.l.* females to species, and to measure the prevalence of *kdr* and *ace-1* resistance mutations.

In addition to the HLC sampling, CDC light traps will be placed every other month in 12 randomly selected houses for one night in each village. These traps will be set in the sleeping area at 18:00 and removed the following morning at 08:00. The study team members will record the number of people who slept in the room during the trap night, the number of LLINs installed in the sleeping area, and whether the people reported sleeping under the available LLINs during the trap night. The mosquitoes captured in the traps will be brought back to the laboratory for morphological species identification.

To monitor resistance in the study area throughout the course of the trial, larval dips will be used to collect *An. gambiae* larvae from eight study villages (four per arm). These field-collected larvae will be reared to adulthood in the insectary at IPR, and bioassays will be conducted with 2–5 day old females. WHO insecticide susceptibility bioassays will be conducted using diagnostic concentrations of the active ingredient in the LLINs (deltamethrin) and the eave tubes (beta-cyflutherin). CDC bottle bioassays will also be done with both actives, to measure resistance intensity. A susceptible *An. gambiae* lab strain (Kisumu) will be tested as a control in these bioassays. After the bioassays, surviving mosquitoes will be put in RNAlater and stored at -80 °C for subsequent microarray analysis to monitor expression of genes potentially involved in metabolic resistance.

### Social science

During the trial, data will be collected on perceptions of and willingness to adopt the SET intervention, knowledge relating to the SET intervention, and attitudes and emotions relating to malaria and new malaria prevention interventions. These data will be collected using three complimentary methodologies: (1) questionnaires administered to homeowners and heads of household, (2) ethnographic studies (observation and informal discussion), and (3) focus groups.

The questionnaires will be administered before the installation of the intervention and at the end of the monitoring period in the six villages designated for social science activities (see [33] for more details on the methodology for these activities). Questionnaires will also be administered in two of the villages midway through the monitoring period. The ethnographic studies will be conducted during and directly after the SET installation process, and during the installation of insecticide treated inserts in three villages in the SET arm. Focus group discussions will be held midway through the monitoring period with people drawn from diverse demographic groups (mothers, village leaders, young adults) in two SET + LLIN villages and one LLIN village.

### Economics

Data on the incremental financial and economic costs of SET will be collected alongside the intervention and attributed to either housing modification (screening, closing eaves), eaves tube installation, SET maintenance, or treatment and retreatment of inserts with insecticide. Where resources (e.g. staff) are shared between more than one element, costs will be allocated using a suitable proxy. Costs for research activities will be excluded. Financial costs will be obtained from project expenditure records. Economic costs (including financial expenditure and donated resources) will be identified from project records and via social science activities, with a value for donated resources being imputed from market rates. Capital costs will be annualized over their useful life (financial costing) and annualized at a discount rate of 3% in the economic costing.

The numbers of malaria cases averted in the SET + LLIN arm compared to the LLIN only arm will be used to calculate disability adjusted life years (DALYs) averted using standard methods [34]. If the epidemiological data suggest an effect on anemia and/or respiratory infections, this will also be included in the DALY calculations.

### Trial oversight

A Trial Steering Committee (TSC) will be established to provide oversight of the study. TSC members will be independent of the trial and its institutions, and have the necessary expertise to monitor study progress and participant safety. The TSC will be responsible for monitoring the progress of the trial, adherence to protocol, patient safety, and for consideration of new information. The TSC will also have access to the study data at regular intervals to determine if additional interim analyses of trial data should be undertaken, and assess any safety issues that may arise during the study. The TSC can make the decision to stop the study at any point.

Day-to-day management of the trial is the responsibility of the chief investigator and co-investigators. They will maintain appropriate medical and research records, in compliance with Good Clinical Practice (GCP), and all regulatory and institutional requirements. Standard Operating Procedures (SOPs) will be developed in accordance with Good Clinical Practice (GCP) and Good Field Entomology Practice (GFEP). An independent consultant will review study protocols, SOPs, and QA reports. A study monitor will conduct audits at key phases of the study.

### Safety considerations

The LLINs distributed as part of this trial (Permanet 2.0) have been fully evaluated by the WHO Pesticide Evaluation Scheme (WHOPES) and are routinely distributed by the NMCP in Côte d’Ivoire.

The beta-cyfluthrin used on the eave tube inserts is a pyrethroid insecticide, which typically have low mammalian toxicity and good safety profiles. The trial will use a commercially available formulation of the insecticide. The inserts will be machine treated to minimize contact with the insecticide dust. All personnel responsible for treating and installing treated inserts will be provided with the appropriate personal protective equipment (PPE).

In addition to safety considerations relating to the use of insecticides, concerns have previously been raised with regards to house modifications and the possibility that they might increase risk of acute respiratory infections due to changes in the indoor environment [12]. To monitor for adverse events (AEs), regardless of whether they are due to insecticide use or house modifications, children enrolled in the ACD cohort will be monitored at each visit for symptoms including skin irritation, respiratory problems, headaches, fatigue, and nausea. The nurses responsible for the ACD visits will record these or any other adverse events, and report them to their supervisor. AEs occurring in non-cohort children living in the study villages will be recorded through passive monitoring of visits to the local health facilities and the CHWs. If a parent or guardian agrees to participate, a verbal autopsy using an established questionnaire will be conducted after any deaths in the cohort. The host institution (IPR) medical expert and his clinical team will be responsible for recording, reporting, and managing AEs and SAEs, including follow-up, in accordance with national guidelines. A report on AEs and SAEs, including deaths in the cohort, will be provided regularly to the TSC.

HLC capturers will be vaccinated against yellow fever free of charge if they do not already have proof of yellow fever vaccination. If they become sick with malaria at any point during the trial, they will be provided treatment free of charge at the nearest health facility.

### Handling of drop-outs / withdrawals

Participants are free to withdraw from the study at any point for any reason. Participants include children in the ACD cohort, households where entomological monitoring is conducted, and capturers conducting HLCs. For children in the ACD cohort, the reason for premature termination or withdrawal will be documented – e.g., SAE, withdrawal of consent, loss to follow up. If the child withdraws from the cohort for any of these reasons, another child will be selected to replace them, preferably in the same age group and from the same household but if that is not possible, then from another randomly selected household.

## Study endpoints

### Epidemiological

The primary endpoint of the trial will be the incidence of clinical malaria determined by active case detection in children six months to ten years of age. Clinical malaria will be defined as an axillary temperature of ≥37.5 °C combined with a positive mRDT. The secondary clinical endpoints will be: (1) incidence of asymptomatic malaria infection detected by microscopy and/or PCR, (2) prevalence of anemia in children five years of age or younger (measured four times during the study), and (3) incidence of the following symptoms of respiratory infection: cough, nasal discharge, rales, elevated age specific respiratory rate, and chest indrawing.

### Entomological

The primary entomological endpoints will be the mean number of malaria vectors (*An. gambiae s.l.*, *An. funestus*, and *An. nili*) captured per person per night both indoors and outdoors by HLC, and the mean number of malaria vectors captured indoors per trap per night by CDC light trap. The secondary endpoints will be: (a) the entomological inoculation rate (EIR) for *An. gambiae s.l.*, *An. funestus*, and *An. nili* calculated as the mean number of sporozoite infective bites/person/year, (b) parity of *An. gambiae s.l.*, *An. funestus*, and *An. nili*, (c) the prevalence (% mortality using a diagnostic dose) and strength (LD50 and resistance ratios) of insecticide resistance to deltamethrin and beta-cyfluthrin measured before, during, and after the trial period in *An. gambiae s.l*.

### Social science

The social science endpoints will be the percentage of homeowners who consent to the SET intervention when offered, and the degree to which consent can be predicted based on psychosocial predictors. In addition, observations and discussions on attitudes towards the technology will be considered in anticipation of diffusion and scale up of the technology to other communities.

### Economics

The economic endpoints will be marginal economic and financial cost per malaria case averted and the cost per DALY averted of the SET intervention compared to LLINs alone.

## Sample size rationale

### Epidemiological

The epidemiological impact of SET alone or in combination with LLINs is currently unknown. A minimum effect size of 40% was used in the sample size calculations, which is similar to the effect size used in other sample size calculations for house-based malaria interventions [12, 35]. The mean incidence of clinical malaria in the reference arm was assumed to be 0.5 malaria cases per child per year, based on existing data from the study area. Further assuming a coefficient of variation (k) of 0.5, (a conservative estimate for a high transmission setting), 80% power and significance level of 5%, requires 20 clusters per arm and 50 children per cluster to be followed for two years (encompassing 2 peak transmission seasons).

### Entomological

Pilot studies in semi-field systems in Tanzania and Kenya suggest an up to 80% reduction in mosquito entry into houses with eave tubes and screening [20, 21]; a more conservative 50% reduction was used for these sample size calculations. Again, this is consistent with other RCTs testing the effect of house modifications on entomological endpoints [12, 13]. A baseline survey conducted during the dry season in the study area had mean HLC catches of 23.5 *An. gambiae*/trap/night, with SD = 17 and intraclass correlation (ICC) = 0.84. For 80% power to detect a 50% drop in mean mosquito densities of 23 mosquitoes at the 5% significant level, 7 households need to be sampled in each of the 40 villages. To capture the substantial seasonal changes and to accommodate available resources, 4 randomly selected households will be sampled over one night in each village every month, powered to detect a difference for every two months of pooled data.

CDC light traps consistently caught fewer mosquitoes than the HLC during the baseline survey, with a mean nightly catch of 2.5 *An. gambiae*, a log SD of 1.7 and ICC = 0.229. To detect a 50% reduction mosquito density with 80% power at the 5% significance level requires 12 households per cluster. In order to capture seasonal variation in mosquito density, collections will be performed every two months.

## Data management

Clinical data will be collected by study nurses on Android tablets using standardized data entry forms in the Open Data Kit (ODK) Collect app and sent directly to a secure electronic server. Routine data checks will be performed to identify incomplete, missing, inaccurate, or inconsistent data, which will be rectified by the appropriate supervisor. Field workers will not have access to the data once it has been collected. Children in the ACD cohorts will be assigned a unique code, which will be used as an anonymous identifier in the datasets and to label clinical samples. Access to the data will be password protected and restricted to only authorized study investigators and data management staff. Any hardcopy documents will be stored in locked filing cabinets and accessible only to authorized study personnel. Documents will be held for at least five years at the host institution (IPR).

## Statistical analysis

### Epidemiological

Protective efficacy against clinical malaria and malaria infection will be determined by comparing incidence rates of malaria infection and clinical malaria between arms. Following any treatment for malaria, a child will not be considered at risk for four weeks and this period will therefore be censored from follow-up. Overnight stays outside of the village will be recorded with a short questionnaire administered during the cohort visits. If travel outside of the village is common, it will be included as a covariate in the analysis.

Primary intention to treat analysis will be comparison of the incidence of clinical malaria episodes in the two intervention groups. All analysis will use a mixed effects model to test the difference in incidence rate between the two arms, allowing for repeated measurements on the same individual, and within-cluster correlation of responses. The effect of year and possible confounders such as age of child, gender, antimalarial drug use, baseline infection status, etc. will be analyzed in separate as per protocol analysis.

Survival analysis will be used to compare time to first infections, adjusting for confounding factors using a Cox proportional hazards model, again using methods that allow for within-village correlation of responses.

### Entomological

Differences in mean mosquito density (for indoor and outdoor catches) between the two study arms will be analyzed using Poisson regression or negative binomial models, accounting for within-village correlation of responses. Sporozoite rates will be compared between the two arms and between indoor and outdoor caught mosquitoes, using logistic regression models. EIR will be calculated for each study night and compared between study arms using negative binomial regression.

### Social science

Pre-intervention willingness to adopt the SET intervention will be compared to actual consent by logistic regressions. The predictors of willingness to adopt will be tested with structural equation modeling. Profiles of perceptions of the SET intervention will be identified using latent class analysis. Ethnographic and focus group information will be evaluated using thematic analysis.

### Economics

The economic and financial cost of the SET intervention will be presented as total and disaggregated by program element to illustrate the relative share of each element to program costs. Costs will also be presented according to cost categories (e.g. labor, insecticide) to illustrate cost drivers. Costs will be converted into cost per house and cost per person receiving the intervention per year, to facilitate comparison with other malaria vector control interventions (e.g. IRS). Alternative program scenarios (e.g. different scale, duration and insecticide treatment frequency) will be presented to estimate operational implementation costs. Uncertainty and sensitivity analysis will be conducted.

The numbers of malaria cases averted in the SET + LLIN arm, compared to the LLIN only arm, will be used to calculate disability adjusted life years (DALYs) averted using standard methods [34]. If the epidemiological data suggest an effect on anemia or respiratory infections, this will also be included in the DALY calculations. Together with cost data, DALY estimates will be used to compare SET with other potential malaria interventions using evidence from the literature.

### Interim analysis

An interim analysis of malaria incidence and transmission potential will be done after one year of monitoring. The purpose of the interim analysis is to inform planning of future trials. The current protocol will remain unchanged regardless of interim results. The funder will not have access to any data, only summary results. P-values will be adjusted appropriately in the final analysis to account for the interim analysis.

## Discussion

Past success in reducing the global malaria burden has highlighted both the importance of vector control in preventing transmission, and the challenges that remain in meeting the targets set out by the WHO Global Technical Strategy (GTS) for Malaria 2016–2030 [3]. IRS coverage has declined to just 2.9% of people at risk for malaria worldwide, likely due to the increased cost associated with switching away from pyrethroid insecticides in response to widespread pyrethroid resistance in vector populations [8]. This leaves the majority of malaria vector control dependent on LLINs. While LLIN coverage has increased over time, there are still concerns over operational limits on increasing coverage and use [36, 37] and insecticide resistance in vector populations [38, 39]. Even maintaining current levels of LLIN coverage might be insufficient to prevent a resurgence in case incidence, as immunity wanes in populations following LLIN distribution campaigns [40]. Thus, there is a strong argument to be made that meeting the GTS milestones will require a greater diversity of vector control tools than currently exist [4, 5].

The aim of this CRT is to evaluate whether SET provides additional protection against malaria, compared to LLINs alone. Based on previous house improvement trials [11, 13, 14], “mosquito proofing” of houses (screening windows, sealing cracks and open eaves) is expected to reduce the number of infective bites received indoors and, consequently, reduce malaria incidence in members of households with the SET intervention. It is also possible that the insecticide component of the SET intervention could have an additional effect on malaria incidence in the SET villages, by killing mosquitoes that attempt to enter houses such that infective bites are reduced even in unmodified houses [19, 41]. However, the trial is not explicitly designed to disaggregate the protection provided by the house modifications and the protection provided by the insecticide.

The decision was made to use a pyrethroid insecticide for this CRT, despite intense pyrethroid resistance in the study area. The rationale for this decision was two-fold: first, pyrethroids are a relatively safe class of insecticides with low mammalian toxicity and second, of all the candidate actives evaluated in preliminary testing, the beta-cyfluthrin formulation had the highest bioassay mortality for the longest period of time when tested with local, field collected, pyrethroid resistant mosquitoes (Oumbouke, unpublished observations). The efficacy of a pyrethroid against resistant mosquitoes is not surprising, given that the delivery method (i.e. a powder formulation on electrostatic netting) has previously been shown to be effective against resistant mosquitoes [23]. Although a pyrethroid is being used for the current CRT, this study partly serves as a proof of concept for the SET intervention as a delivery mechanism. If it is effective, SET could potentially be implemented with a variety of other active ingredients and formulations, including non-pyrethroid insecticides and even biological actives such as entomopathogenic fungi [42, 43].

**Fig. 3:**
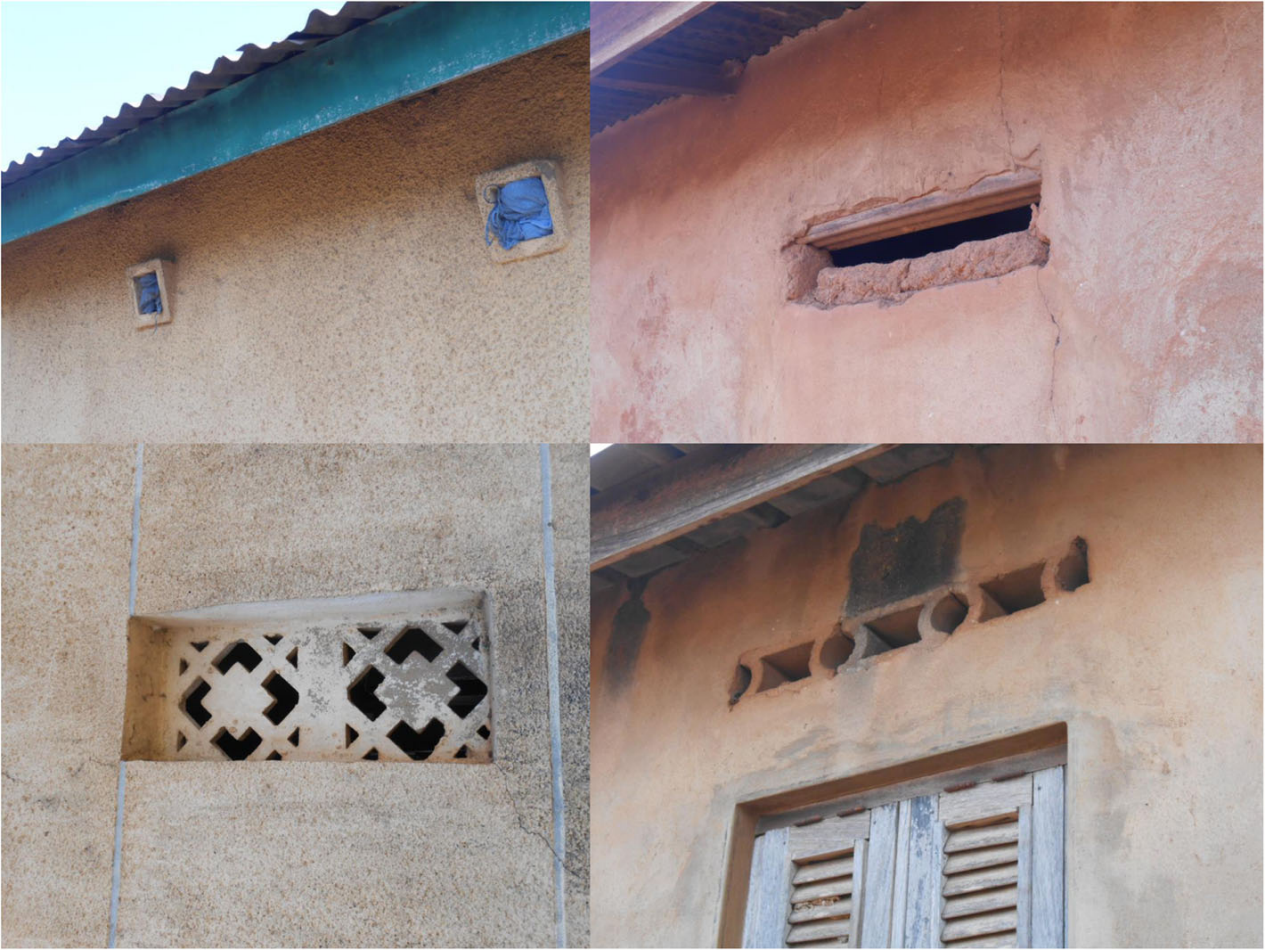
Examples of ventilation bricks commonly found in the study area in central Côte d’Ivoire. These are often either left unscreened, or blocked (e.g. with LLINs, as shown in the top left image)

Given that SET requires permanent modifications to the house structure, both cost and user acceptability are a concern. Several social science and economic activities have been planned in parallel with the epidemiological and entomological monitoring to address these issues. One specific concern is that closing open eaves could reduce airflow within the house, which could alter indoor temperature and humidity, and thus negatively impact the comfort of those living in the house. It is worth noting that the majority of houses in this area already have closed eaves, in many cases with ventilation holes built in at eave level (Fig. 3), similar to eave tubes. However, there are regional differences in housing design, and the feasibility and acceptability of the SET intervention is likely to depend on local context, which will require further studies with the technology.

If the SET intervention is effective at reducing malaria, acceptable to the local population, and cost-effective as a malaria prevention intervention, the question becomes how to implement the intervention at scale. It has been argued that house improvements on their own will likely have to be implemented outside of the existing health sector, which provides funding for LLIN distributions and IRS campaigns [12]. However, in addition to house improvements, the SET intervention includes a mosquito-killing device in the eave tube, which could provide one avenue for a more typical, donor-funded distribution model. This does not have to be the only model, but rather one of a suite of implementation strategies. This CRT serves as a first, essential step in demonstrating the epidemiological and entomological impact of the SET intervention, and estimating acceptability and cost-effectiveness to inform whether it is indeed a viable malaria control strategy that should be implemented at scale.

## Abbreviations

ACD: Active case detection
AE: Adverse events
CDC: Centers for Disease Control
CHW: Community health workers
CRT: Cluster randomized controlled trial
DALY: Disability-adjusted life year
EIR: Entomological inoculation rate
GCP: Good clinical practice
GFEP: Good field entomology practice
GTS: Global technical strategy
HLC: Human landing catch
IPR: Institut Pierre Richet
IRS: Indoor residual spraying
LLIN: Long-lasting insecticidal nets
mRDT: Malaria rapid diagnostic test
NMCP: National malaria control program
RCT: Randomized controlled trial
SAE: Serious adverse events
SET: Screening plus eave tubes
SOP: Standard operating procedure
TSC: Trial steering committee
WHO: World Health Organization
